# Supplementary material for: No relationship between frontal alpha asymmetry and depressive disorders in a multiverse analysis of five studies
Source: eLife. 2021 May 26;10:e60595. doi: 10.7554/eLife.60595 (PMC8154036; doi:10.7554/eLife.60595)
Supplement: Transparent reporting form [file elife-60595-transrepform.pdf]

eLIFE
1st Floor
24 Hills Road
Cambridge CB2 1JP, UK
P 01223 855340
W elifesciences.org
T @elife

eLife's transparent reporting form

We encourage authors to provide detailed information *within their submission* to facilitate the interpretation and replication of experiments. Authors can upload supporting documentation to indicate the use of appropriate reporting guidelines for health-related research (see EQUATOR Network), life science research (see the BioSharing Information Resource), or the ARRIVE guidelines for reporting work involving animal research. Where applicable, authors should refer to any relevant reporting standards documents in this form.

If you have any questions, please consult our Journal Policies and/or contact us: editorial@elifesciences.org.

Sample-size estimation

- You should state whether an appropriate sample size was computed when the study was being designed
- You should state the statistical method of sample size computation and any required assumptions
- If no explicit power analysis was used, you should describe how you decided what sample (replicate) size (number) to use

Please outline where this information can be found within the submission (e.g., sections or figure legends), or explain why this information doesn't apply to your submission:

In each study the decision on the sample size was based on common sample sizes reported in previous literature on alpha asymmetry in depression. Two of the studies used in our paper were recorded in the past, when sample size estimation was not common. It should also be noted that recruiting depressed participants is a difficult and lengthy process, and the final sample size is an effect of cost-benefit analysis (also taking into account project length and available funding). Two other datasets we use in the paper (Study IV and V) are publicly available data and the size of the groups did not depend on our judgement.
We overcome the sample size limitations of single studies by presenting results for all five studies in a multiverse approach.

Replicates

- You should report how often each experiment was performed
- You should include a definition of biological versus technical replication
- The data obtained should be provided and sufficient information should be provided to indicate the number of independent biological and/or technical replicates
- If you encountered any outliers, you should describe how these were handled
- Criteria for exclusion/inclusion of data should be clearly stated
- High-throughput sequence data should be uploaded before submission, with a private link for reviewers provided (these are available from both GEO and ArrayExpress)

Please outline where this information can be found within the submission (e.g., sections or figure legends), or explain why this information doesn't apply to your submission:

eLife Sciences Publications, Ltd is a limited liability non-profit non-stock corporation incorporated in the State of Delaware, USA, with company number 5030732, and is registered in the UK with company number FC030576 and branch number BR015634 at the address 1st Floor, 24 Hills Road, Cambridge CB2 1JP | August 2014

The exclusion criteria for subjects are detailed in the *Methods - Participants* section while the exclusion criteria for channels, signal segments and ICA components are described in *Methods - Data preprocessing* section.

Statistical reporting

- Statistical analysis methods should be described and justified
- Raw data should be presented in figures whenever informative to do so (typically when N per group is less than 10)
- For each experiment, you should identify the statistical tests used, exact values of N, definitions of center, methods of multiple test correction, and dispersion and precision measures (e.g., mean, median, SD, SEM, confidence intervals; and, for the major substantive results, a measure of effect size (e.g., Pearson's r, Cohen's d)
- Report exact p-values wherever possible alongside the summary statistics and 95% confidence intervals. These should be reported for all key questions and not only when the p-value is less than 0.05.

Please outline where this information can be found within the submission (e.g., sections or figure legends), or explain why this information doesn't apply to your submission:

Signal processing and statistical analysis methods were described in sections: *Signal analysis* and *Statistical analysis.*
We presented the results with the required details in section *Results* and *Supplementary materials*, figures (*Figures 3 - 7*) and tables (*Tables 1 - 8*). The effect size and its 95% confidence interval is given only for single channel pair analyses (*Table 1 and 2; Figure 8 and Figure 8- supplement 1*) because there is no common standard for calculating and reporting effect sizes and confidence intervals for cluster based permutation test results, especially when no clusters were found.

(For large datasets, or papers with a very large number of statistical tests, you may upload a single table file with tests, Ns, etc., with reference to sections in the manuscript.)

Group allocation

- Indicate how samples were allocated into experimental groups (in the case of clinical studies, please specify allocation to treatment method); if randomization was used, please also state if restricted randomization was applied
- Indicate if masking was used during group allocation, data collection and/or data analysis

Please outline where this information can be found within the submission (e.g., sections or figure legends), or explain why this information doesn't apply to your submission:

We described above information in section: *Methods - Participants.*

Additional data files ("source data")

- We encourage you to upload relevant additional data files, such as numerical data that are represented as a graph in a figure, or as a summary table
- Where provided, these should be in the most useful format, and they can be uploaded as "Source data" files linked to a main figure or table
- Include model definition files including the full list of parameters used

eLife Sciences Publications, Ltd is a limited liability non-profit non-stock corporation incorporated in the State of Delaware, USA, with company number 5030732, and is registered in the UK with company number FC030576 and branch number BR015634 at the address 1st Floor, 24 Hills Road, Cambridge CB2 1JP | August 2014

- Include code used for data analysis (e.g., R, MatLab)
- Avoid stating that data files are "available upon request"

Please indicate the figures or tables for which source data files have been provided:

Due to the fact that in our article we conduct many analyses we included supplemental figures with legends to all results in the section *Supplemental Figures*.
The code used for data analysis and visualisation is publicly available on Github (https://github.com/mmagnuski/DiamSar). Information about other python packages used in the analyses is included in *Methods* section and relevant entries are present in the *References*. The data for the three studies are deposited to Dryad repository.

eLife Sciences Publications, Ltd is a limited liability non-profit non-stock corporation incorporated in the State of Delaware, USA, with company number 5030732, and is registered in the UK with company number FC030576 and branch number BR015634 at the address 1st Floor, 24 Hills Road, Cambridge CB2 1JP | August 2014
